# Phytochemistry and Pharmacological Properties of Medicinal Plants

**DOI:** 10.3390/plants15081220

**Published:** 2026-04-16

**Authors:** Alfred Maroyi

**Affiliations:** Department of Biotechnology and Biological Sciences, University of Fort Hare, Alice 5700, South Africa; amaroyi@ufh.ac.za

Medicinal plants have a long history of usage in the treatment and management of human and animal diseases and ailments. About 80% of people in both developed and developing countries depend on traditional medicines for their primary health care needs [1]. The therapeutic value of medicinal plants and their effectiveness in the treatment and management of human and animal diseases and ailments have been recognized around the world among different traditional medicinal systems such as those of Africa, North Africa, the Middle East, China, India (such as Ayurvedic medicine), Europe, America, Australia and Southeast Asia [2]. Traditional medicines, plant-derived medicines, other natural products and their derivatives are becoming increasingly popular in modern society as natural alternatives to synthetic chemicals [2]. Increasing acceptance of traditional medicine, plant-derived medicines and other natural products is often attributed to their deep-rooted cultural significance, affordability, and accessibility to the larger population in both developed and developing countries. This heavy reliance of local communities on traditional medicine reflects both the historical development of the Indigenous or traditional pharmacopeia and complex and dynamic human–nature relationships. Similarly, in other countries like South Africa, the use of medicinal plants and their extracts are an important component of the traditional pharmacopeia or traditional *Materia medica* and are also an aspect of the daily lives of many local people and an essential part of the South African cultural heritage [3]. In this context, knowledge about the identity of plant species used as sources of traditional medicines is important, as well as their medicinal applications, the plant parts used, and the dosage and administration. Pharmaceutical research, therefore, has brought about major shifts in perceptions of traditional medicine and significant changes concerning how this natural resource is utilized. This means that medicinal plants which historically were available only as bark, bulbs, leaves, roots, fruits, stems, rhizomes, tubers, corms, flowers, seeds, gums, exudates and nectar are today herbal medicines that are subjected to thorough ethnopharmacological research and development. The quality of a herbal medicine is directly related to its active secondary metabolites, commonly known as its phytocomplex [4]. A phytocomplex is a mixture of active and inactive molecules extracted from medicinal plant species characterized by specific biological activities and designed to harness the synergistic effects of all the molecules rather than a single isolated molecule. The biological activities of a phytocomplex are, therefore, stronger than the sum activities of a single active molecule [4]. Previous research has shown that phytocomplexes are characterized by synergy, several and various mechanisms of action, better bioavailability, minor toxicity and better tolerance [5]. In herbal medicine and phytotherapy research, phytoequivalence is often used to verify that a phytocomplex from a medicinal plant matches that of a clinically validated product [6]. Phytoequivalence is the concept that two herbal extracts are equivalent if they share matching phytochemical compounds and identical biological activities [7]. Chromatographic techniques such as high-performance thin-layer chromatography (HPTLC) are often used to establish similarities between herbal extract profiles including in vitro or in vivo studies for efficacy [7].

The therapeutic applications of medicinal plants are often linked to their varied phytochemical properties. Numerous pharmaceutical drugs that are currently utilized as therapeutic agents and form the cornerstone treatments in contemporary clinical practice have been derived from natural products, particularly medicinal plants. Therefore, natural products serve as crucial sources of bioactive compounds that are therapeutically important and essential in pharmaceutical drug discovery research [8,9]. Well-known examples of plant derived medicines, plant drugs, phytomedicines or phytopharmaceuticals include artemisinin (*Artemisia annua* L.), codeine and morphine (*Papaver somniferum* L.), colchicine (*Colchicum autumnale* L.), combretastatin (*Combretum caffrum* (Eckl. & Zeyh.) Kuntze), digoxin (*Digitalis lanata* Ehrh.), paclitaxel (*Taxus brevifolia* Nutt.), reserpine (*Rauvolfia serpentina* (L.) Benth. ex Kurz, silymarin (*Silybum marianum* (L.) Gaertn.), vinblastine and vincristine *Catharanthus roseus* (L.) G.Don [3]. These phytomedicines, natural products and their derivatives provide the basis for more than 25% of all pharmaceutical drugs in clinical use in the world [3]. Therefore, an important and essential step aimed at evaluating the therapeutic significance of herbal medicine decoctions, infusions, syrups, juices, tinctures, steam inhalations and steam baths, powder snuffs, enemas, poultices, gargles, ointments, lotions, nasals and eyedrops involves isolation and identification of the pharmacologically active constituents of such medicinal plants, and this research approach forms the foundation of modern pharmaceutical development. This innovative phytopharmaceutical research coupled with state-of-the-art technologies has validated many of the healing attributes for which individual medicinal plant species have long been used traditionally. In general, all phytomedicines must have some pharmacological activity in order to be of therapeutic benefit for a particular disease or ailment [10].

Typically, many plant-based traditional medicines and natural products are accompanied by evidence-based biological efficacies, but there is often inadequate information about their standardized quality, mode of action, toxicology, how they interact with western medicines, evidence-based safety and regulatory approvals of these plant-based therapies [1,8,10,11]. The increased interest, trade and use of plant-based therapies and natural products prompted the World Health Organization [12] to draft internationally recognized guidelines for the quality assessment of herbal products, wherein governments of different countries are encouraged to promote the use of herbal medicines that have been scientifically validated for quality, safety and efficacy. Hence the need for pharmacological validation of plant-based traditional medicines and natural products through in vitro and in vivo and clinical studies. Clinical studies are the most convincing scientific method of proving the safety and efficacy of plant-based therapies and natural products [2].

Collectively, the contributions in this Special Issue reflect the diversity and depth of current research in phytochemistry and the pharmacological properties of medicinal plants. This Special Issue delves into the medicinal uses, phytochemical characterization, and pharmacological and toxicological properties of medicinal plants. Therefore, this compilation stimulates further research into traditional systems of medicine and how and why the plant species are used as herbal medicines, exploring their active phytochemical compounds and pharmacological properties using various in vitro and in vivo models, safety studies and clinical validation.
